# What does high and low have to do with it? Performance classification to identify health system factors associated with effective prevention of mother-to-child transmission of HIV delivery in Mozambique

**DOI:** 10.7448/IAS.17.1.18828

**Published:** 2014-03-24

**Authors:** Sarah Gimbel, Joachim Voss, Alison Rustagi, Mary Anne Mercer, Brenda Zierler, Stephen Gloyd, Maria de Joana Coutinho, Maria de Fatima Cuembelo, Kenneth Sherr

**Affiliations:** 1 Department of Family and Child Nursing, University of Washington, Seattle, WA, USA; 2 Department of Global Health, University of Washington, Seattle, WA, USA; 3 Health Alliance International, Seattle, WA, USA; 4 Department of Biobehavioral Nursing and Health Systems, University of Washington, Seattle, WA, USA; 5 Health Alliance International, Beira, Mozambique; 6 Department of Community Health, University of Eduardo Mondlane, Maputo, Mozambique

**Keywords:** implementation science, performance measures, PMTCT, Mozambique, performance classification, health systems strengthening

## Abstract

**Introduction:**

Efforts to implement and take to scale highly efficacious, low-cost interventions to prevent mother-to-child HIV transmission (pMTCT) have been a cornerstone of reproductive health services in sub-Saharan Africa for over a decade. Yet efforts to increase access and utilization of these services remain far from optimal. This study developed and applied an approach to systematically classify pMTCT performance to identify modifiable health system factors associated with pMTCT performance which may be replicated in other pMTCT systems.

**Methods:**

Facility-level performance measures were collected at 30 sites over a 12-month period and reviewed for consistency. Five combinations of three indicators (1. HIV testing; 2. CD4 testing; 3. antiretroviral prophylaxis and combined antiretroviral therapy initiation) were compared including a composite of all three, a combination of 1. and 3., and each individually. Approaches were visually assessed to describe facility performance, focusing on rank order consistency across high, medium and low categories. Modifiable and non-modifiable factors were ascertained at each site and ranking process was reviewed to estimate association with facility performance through unadjusted Chi-square tests and logistic regression. After describing factors associated with high versus low performing pMTCT clinics, the effect of inclusion of the 10 middle performers was assessed.

**Results:**

The indicator most consistently associated with the reference composite indicator (HIV testing, antiretroviral prophylaxis and combined antiretroviral therapy) was the single measure of antiretroviral prophylaxis and combined antiretroviral therapy. Lower performing pMTCT clinics ranked consistently low across measurement strategies; high and middle performing clinics demonstrated more variability. Association between clinic characteristics and high pMTCT performance varied markedly across ranking strategies. Using the reference composite indicator, larger catchment area, higher number of institutional deliveries, onsite CD4 point-of-care capacity, and higher numbers of nurses and doctors were associated with high clinic performance while clinic location, NGO support, women's support group, community linkages patient-tracking systems and stock-outs were not associated with high performance.

**Conclusions:**

Classifying high and low performance provided consistent results across ranking measures, though granularity was improved by aggregating middle performers with either high or low performers. Human resources, catchment size and utilization were positively associated with effective pMTCT service delivery.

## Introduction

Sub-Saharan Africa bears a disproportionate HIV burden with 92% of HIV-positive pregnant women and 90% of all incident HIV cases in children globally [1]. Efforts to implement and take to scale highly efficacious, low-cost interventions to prevent mother-to-child HIV transmission (pMTCT) have been a cornerstone of reproductive health services in high-burden HIV settings for over a decade, leading to substantial increases in access and utilization of preventive services, with the objective of prevention of paediatric HIV infection. However, pMTCT services in sub-Saharan Africa remain sub-optimal, with an estimated 56% of HIV-positive pregnant women accessing pMTCT services in 2012 [2]. Parallel to efforts to expand pMTCT, efforts have focused on implementing more efficacious antiretroviral (ARV) prophylaxis (PPO) regimens, which together have reduced HIV transmission to newborns [3, 4].

To translate more efficacious regimens to effective pMTCT services, heterogeneous clinic-level facilitators and barriers that impact access and utilization must be addressed. By identifying modifiable health system factors associated with high and low-performing pMTCT systems, best practices can be disseminated and implemented to maximize the number of women and children reached with effective pMTCT services. Likewise, identified factors associated with poorer performance can be addressed across facilities to reduce their replication.

PMTCT is a highly complex package of healthcare interventions that spans multiple stages of women's reproductive and children's developmental lifespan, and health services specific to these stages. To maximize the benefit to mothers and children, pMTCT is delivered in a timed sequence, or “cascade” that includes: attendance at antenatal care (ANC), HIV counselling and testing, provision of combined antiretroviral therapy (cART) – previously prophylactic antiretrovirals (ARVs) – safe delivery, administration of prophylactic ARVs for the exposed newborn through the breastfeeding period, safe infant feeding, infant follow-up including HIV testing, family planning and linkages to long-term HIV care and treatment [5, 6]. Comprised of a sequence of linked events, the pMTCT cascade provides a useful framework for organizing pMTCT services and quantifying attrition along the pathway from identifying HIV-positive women, preventing HIV transmission to children and ensuring long-term access to HIV care and treatment [7]. Improving pMTCT effectiveness requires increasing the number of women and their children who successfully pass through the multiple, sequential steps in the pMTCT cascade; therefore identifying replicable approaches to optimize the cascade is a critical priority [8].

### Methodologies to classify and learn from differential performance

Identifying high and low-performing health facilities, and the drivers of performance, may highlight best practices associated with effective pMTCT service delivery in resource-limited settings for replication in other pMTCT systems. Two methodologies developed by systems management and behavioural sciences that utilize performance classification and exploit performance differentials to improve health system functioning and individual-level health behaviours include Lot Quality Assurance Sampling (LQAS) and Positive Deviance [9–12].

LQAS includes the inspection of a small representative sample of a production lot, and if the number of defective goods in the sample exceeds a predetermined allowable number, the lot is rejected; otherwise the lot is classified as having acceptable quality [13].

LQAS has been applied to the health sector to conduct rapid assessments of service coverage, the accuracy of health records, logistics and supply chain performance, training programs and the quality of facility management [10, 14–16]. LQAS identifies low performers, so managers can target their support to sub-optimally performing geographic areas or facilities, and provide insights into the relationship between improvement strategies and their effectiveness. However, beyond identifying failing production systems in specific geographic or service areas, LQAS does not provide guidance on which components of the production system are failing [17] Thus, it has limited utility for complex interventions like pMTCT, which may require tailored interventions depending on which pMTCT cascade step is under-performing.

On the other side, positive deviance focuses on high performers to understand and amplify their traits, practices and characteristics across a broader population [18]. By identifying successful individuals, families or groups with uncommon, beneficial practices (positive deviants) – and modelling these practices for others – positive deviance intends to build on internal solutions that are potentially more culturally appropriate and sustainable [19]. Positive deviance has been applied to multiple interventions in the areas of nutrition, hygiene, obesity prevention, health service utilization, breastfeeding adherence and HIV/AIDS prevention [20–27]. By contrasting high and low-performing individuals, positive deviance provides a framework for understanding and improving outcomes in low-performing entities [18, 28].

Despite their targeted use of either high (positive deviance) or low (LQAS) performance, neither adequately articulates a standardized approach for overall performance classification, which is challenging for cascade type services that span multiple arenas. Various methodologies assess the performance and/or effectiveness of pMTCT programs using single measures. Some of these include the uptake of ARV regimens during pregnancy and delivery as measured through program data [29]; the use of cord blood assessments which are able to assess actual ARV coverage for comparison with service data [30]; and the use of early infant diagnosis which is arguably the most accurate measure of this program which is designed to prevent mother-to-child transmission of HIV, as well as to promote early clinical care and continuous follow-up pMTCT for the exposed infant [31]. However, for health managers to view their service as a system, a broader understanding of the pMTCT intervention is needed. Specifically, methodologies which promote a broader vision for health workers must take into account the pMTCT cascade as well as specific health system factors that may influence its effectiveness.

The objective of this study is to develop and explore a performance classification approach for health managers to use to identify modifiable health system factors associated with pMTCT performance. By describing this approach, we provide a novel model for pMTCT performance assessment that relies on routinely collected and reported data, and which can be used by health managers to identify modifiable factors associated with pMTCT performance to inform program improvement efforts.

## Methods

### Study design

Activities were conducted as part of a multi-methods, cross-sectional study designed to identify modifiable and non-modifiable health systems factors associated with pMTCT service performance. The results presented here contributed to the parent study which will be conducted in three sub-Saharan countries by developing the health facility performance ranking approach as a first step for identifying systems-level factors associated with high and low-performing clinics.

### Study setting

Thirty public sector health facilities with pMTCT services in three districts along the Beira corridor (the main transport route connecting the port city of Beira with Zimbabwe) in Sofala province, central Mozambique, were included in the study. Sofala has an estimated population of 1.8 million of which 47% live in the study districts of Beira city, Dondo and Nhamatanda [32]. Sofala province has an estimated adult HIV prevalence of 15.5% [33], which has been consistently higher among women routinely tested for HIV in ANC (17.8%) [34].

Since its introduction in 2002, pMTCT expansion has increased to reach 100% of all public sector clinics with ANC services in the three study districts (and 86% of all facilities nationally) [35]. The gaps in the pMTCT cascade limit its effectiveness resulting in approximately 28% HIV infection rate in infants born to HIV-positive women in 2012 despite high availability of pMTCT and approximately 95% ANC attendance rate [36].

Study facilities included all public sector health facilities that met the inclusion criteria of: 1) location in the three districts; 2) provision of pMTCT at ANC in the last six months; and 3) consent to participate in the study. Two facilities were excluded because they were unwilling to participate or had limited access due to flooding.

### Data sources

Clinic data for pMTCT performance ranking were sourced from provincial program reports covering January–December 2012. Data were based on monthly health facility data, which in parallel is entered into the national health information system at the district level. Monthly facility-level data were assessed for availability by the study team to identify missing reports, and irregular or missing data were crosschecked with facility-level registries to ensure accuracy. Missing data were recovered for all measures except cluster of differentiation 4 (CD4) testing data, which were inconsistently available at the facility-level.

Data on health facility characteristics were collected using a survey developed for study purposes from November 2012 to January 2013 (Supplementary file). Descriptive, facility-level variables were identified through reviews of published literature on pMTCT and quality improvement, and the final list of facility characteristics was developed in consultation with provincial program managers and technical advisors [37–39]. The data collection form was developed in Portuguese and piloted in one facility before study assistants visited all 30 health facilities to collect information from facility managers and front line health workers. Data from each facility were double entered by study personnel to ensure their accuracy.

### Variable definition

#### Performance ranking

Three performance measures were *a priori* selected based on their presence in routine reporting systems and importance for successful pMTCT, including: 1) the proportion of pregnant women in ANC tested for HIV at their first visit; 2) the proportion of pregnant women with a positive HIV test at first ANC visit who had a CD4 test in pregnancy; and 3) the proportion of women with a positive HIV test in the first ANC visit accessing effective PPO or cART in pregnancy (Table 1). HIV testing in the first ANC visit was selected (*versus* testing at any time during pregnancy) because testing in the first ANC is standard as per Ministry of Health (MOH) guidelines, and because an effective pMTCT package should initiate as early in pregnancy as possible [40]. Single-dose nevirapine was not included as an effective pMTCT PPO in the third measure because of its relative ineffectiveness in preventing HIV transmission [41].

**Table 1:** Ranking measures

	Indicator	Pros	Cons
1	% of women with HIV test at first ANC Visit	High availability and standardized collection and reporting across facilities	Little heterogeneity; almost universally high masks performance differences
2	% of pregnant women with positive HIV test at first ANC visit with a CD4 test while in pregnancy	Measures integration across services (e.g. laboratory vs. clinic), high variation across facilities	Data inconsistent or unavailable; cannot link women with outcome to those with HIV test at first ANC visit using routinely reported data
3	% of pregnant women with a positive HIV test at first visit who initiate either bi-/tri-prophylaxis or cART prior to delivery	High availability and standardized collection and reporting across facilities	Cannot link women with outcome to those with HIV test at first ANC visit using routinely reported data

ANC: antenatal care; cART: combination antiretroviral therapy.

The 12 months of pMTCT cascade data were used to develop summary performance scores for each facility (*n*=30). First, each of the three indicators were estimated over a 12-month period (calendar year 2012). These individual indicators were then multiplied to generate the composite indicators. Facilities were ranked into three performance categories (high/middle/low) based on tertiles in the distribution of performance outcomes, rather than using a specific performance threshold level. Visual assessment was then used with one measurement strategy (HIV testing and ARV treatment and ARV PPO) defined as the reference strategy. This strategy was selected as the reference as it was composed of complete data sets and represented multiple steps in the pMTCT cascade.

In order to assess the value of comparing high *versus* low-performing facilities in identifying modifiable health facility characteristics associated with pMTCT performance, we recoded facilities into two additional performance groups – one joining middle and high performers (to compare with low performers), and a second joining middle and low performers (to compare with high performers).

#### Modifiable and non-modifiable facility characteristics

Non-modifiable facility characteristics considered included facility type, classified into three levels – quaternary/tertiary hospitals, secondary hospitals and primary health centres, as well as catchment population sizes which were provided by provincial and district authorities. Additional non-modifiable factors collected included geographic location which was defined as urban/peri-urban/rural based on their location in Beira, Dondo or Nhamatanda municipalities (urban), in the outlying neighbourhoods of Beira (peri-urban), or outside of Beira, Dondo, or Nhamatanda municipalities (rural) and year of pMTCT initiation which was provided by facility leadership.

Modifiable facility level characteristics included staffing which was defined as the number of health workers of cadres most relevant to pMTCT service delivery (physician, non-physician clinician (NPC), maternal and child health nurse, general nurse, midwife, custodian, social worker and activist). Distance to a laboratory with CD4 capacity was also considered modifiable as it could hypothetically be changed if CD4 capacity was introduced through new machines or new PIMA technology. This was estimated using driving distances between health facilities provided by provincial authorities.

Other modifiable factors measures to describe pMTCT organization included integration with laboratory services (number of days per week with CD4 blood draws, availability of on-site laboratory capacity for CD4 and other laboratory monitoring), pharmacy services (medicines distributed via pharmacy or ANC/maternity) and outpatient care (adult and paediatric patient referral and tracking for continuity). Also, modifiable measures of community linkages included the presence of a mothers’ support group, whether community activists carried out patient tracking and whether health workers performed regular community outreach. General management practices were measured by the frequency of staff meetings. A list of essential medicines, supplies and materials related to pMTCT service provision was included in the factors list to assess the availability of key items over the preceding three months, as well as the length of stock outages, and was confirmed using stock registries. These were all deemed modifiable as innovations could be introduced to improve them at the facility, district or provincial level.

Finally overall PMTCT service utilization was captured through four patient volume measures over the six-months before data collection, including the number of ANC consults, the number of postpartum consults, the number of institutional births and the number of modern family planning methods distributed.

### Statistical analysis

To refine the ranking procedures, we first explored whether the rank performance order for the 30 study facilities changed according to performance measurement (including each of the three outcome measures alone, a composite indicator multiplying indicators one and three for each facility (HIV testing and effective PPO or cART), and a composite indicator multiplying indicators one, two and three for each facility (adding CD4 testing to the previous indicator). Visual assessment focused on the consistency of rank order across high, medium and low categories depending on ranking strategy, using the composite indicator of HIV testing and effective PPO or cART as the benchmark, with the top 10 performing sites shaded, the middle 10 in white and the bottom 10 dotted.

Assessment of the impact of ranking process on facility-level characteristics associated with facility performance was carried out for three of the five ranking approaches, excluding CD4 testing data which were found to be less available and reliable. Unadjusted Chi-square tests for independence were performed to estimate the association between performance and health facility characteristics. For continuous variables, logistic regression for performance outcomes was used to quantify the magnitude and statistical significance of any associations with performance.

After describing factors associated with high *versus* low-performing pMTCT clinics, we assessed how the inclusion of the 10 middle-performing clinics affected the list of factors significantly associated with differential pMTCT performance. For this analysis, we developed ordinal logistic regression models including continuous clinic characteristics found to be significant in the bivariate analyses, and examined the magnitude of the associations between three-level (low, middle, high) compared to two-level (low, high, excluding the 10 middle-performing clinics). Next, we aggregated low and middle-performing facilities to compare their characteristics to high-performing facilities, and then aggregated middle and high-performing facilities to compare their characteristics with low-performing clinics. This analysis ranked facilities using indicators one and three (HIV testing at first ANC and receipt of effective PPO or cART), as these measures were most available and measured multiple, essential steps for successful pMTCT.

Data analysis was performed using Stata v11.2 (College Station, TX).

### Ethical approval

Study procedures were approved by the Ethics Committee of the Mozambique MOH and were determined to be non-research by the University of Washington Institutional Review Board.

## Results

The majority of health facilities were public sector, small to mid-sized primary health centres clustered in rural areas along the Beira corridor, with an average catchment population of approximately 28,000 inhabitants (Table 2).

**Table 2:** Characteristics of study slinics

	*N*	%
Clinic location		
Rural	18	60
Urban	5	17
Peri-urban	7	23
Clinic type		
Quaternary/tertiary	1	3
Secondary	1	3
Primary	28	93
Public/private		
Public	27	90
Private	0	0
Mixed	3	10
NGO support		
None	17	57
One	5	17
Multiple	8	26
Year of pMTCT initiation		
Before 2005	5	17
Between 2006 and 2008	21	70
After 2008	4	13
Other characteristics	Mean	Median
Distance to reference laboratory (km)	22	16
Catchment population (people)	27,774	19,644
No. 1st ANC visits in the last 6 months	674	468
No. institutional births in the last 6 months	482	257
No. post-partum visits in the last 6 months	418	189

NGO: non-governmental organization; pMTCT: prevention of mother-to-child-transmission; ANC: antenatal care.

Two hospitals were in the sample, including one quaternary-level referral hospital and one rural hospital. Over half of the facilities reported receiving no non-governmental organization (NGO) support, and almost 70% began pMTCT services between 2006 and 2008, during the push to fully extend pMTCT services to all health facilities with maternal child health (MCH) services nationwide. The mean distance between health facilities and their CD4 reference laboratory was 22 km (13.1 miles).

### Effect of performance measures on ranking

Visual assessment of the effect of performance measures on ranking found a consistency among lower performing clinics, with more variability in rank order among higher performing facilities (Table 3).

**Table 3:** Effect of PMTCT measurement strategy on performance ranking

HIV testing, ART-PPO mother			HIV testing, CD4 testing, ART-PPO mother			ART-PPO mother			CD4			HIV testing		
				
Rank	Clinic	Score	Rank	Clinic	Score	Rank	Clinic	Score	Rank	Clinic	Score	Rank	Clinic	Score
1	A	0.958	1	j	0.745	1	A	0.947	1	J	0.611	1	V	1.00
2	B	0.941	2	E	0.723	2	B	0.923	2	E	0.442	2	Q	1.00
3	C	0.902	3	A	0.705	3	C	0.870	3	N	0.396	3	Z	1.00
4	D	0.864	4	B	0.627	4	I	0.796	4	K	0.310	4	L	1.00
5	E	0.863	5	K	0.620	5	G	0.788	5	A	0.200	5	E	1.00
6	F	0.862	6	N	0.620	6	F	0.788	6	M	0.165	6	O	1.00
7	G	0.857	7	I	0.610	7	H	0.782	7	I	0.124	7	D	1.00
8	H	0.856	8	G	0.603	8	D	0.727	8	G	0.096	8	J	1.00
9	I	0.853	9	C	0.601	9	E	0.726	9	S	0.078	9	K	0.98
10	J	0.813	10	F	0.586	10	M	0.647	10	L	0.056	10	U	0.98
11	K	0.776	11	D	0.576	11	J	0.628	11	BB	0.043	11	A	0.97
12	L	0.761	12	H	0.571	12	K	0.567	12	F	0.034	12	S	0.96
13	M	0.749	13	M	0.554	13	N	0.561	13	R	0.024	13	B	0.96
14	N	0.732	14	L	0.526	14	P	0.547	14	AA	0.007	14	DD	0.95
15	O	0.727	15	S	0.492	15	T	0.545	15	H	0.000	15	Y	0.95
16	P	0.726	16	O	0.484	16	R	0.540	16	Q	0.000	16	F	0.94
17	Q	0.714	17	P	0.484	17	X	0.524	17	Z	0.000	17	C	0.93
18	R	0.713	18	R	0.483	18	L	0.521	18	B	0.000	18	H	0.93
19	S	0.699	19	Q	0.476	19	O	0.453	19	CC	0.000	19	G	0.92
20	T	0.691	20	T	0.461	20	S	0.436	20	O	0.000	20	I	0.91
21	U	0.671	21	U	0.447	21	Q	0.429	21	U	0.000	21	W	0.91
22	V	0.669	22	V	0.446	22	W	0.396	22	W	0.000	22	P	0.91
23	W	0.652	23	W	0.434	23	V	0.367	23	P	0.000	23	N	0.90
24	X	0.640	24	X	0.427	24	Y	0.308	24	T	0.000	24	AA	0.90
25	Y	0.627	25	Y	0.418	25	AA	0.304	25	X	0.000	25	CC	0.89
26	Z	0.615	26	Z	0.410	26	BB	0.275	26	Y	0.000	26	R	0.89
27	AA	0.600	27	AA	0.402	27	Z	0.229	27	DD	0.000	27	BB	0.88
28	BB	0.579	28	BB	0.401	28	CC	0.095	28	V	0.000	28	M	0.85
29	CC	0.494	29	CC	0.329	29	DD	0.000	29	C	0.000	29	T	0.84
30	DD	0.477	30	DD	0.318	30	V	0.000	30	D	0.000	30	X	0.76

Dark grey: high performance; white: middle performance; spotted grey: low performance using dual HIV testing & ART-PPO mother ranking schema; HIV: human immunodeficiency virus; ART: antiretroviral therapy; PPO: prophylaxis; CD4: cluster of differentiation 4.

Of the three indicators representing a single step of the pMTCT cascade, receipt of ARV PPO or cART for women identified as HIV positive at first ANC visit was the most consistent in rank order compared with the reference composite indicator of HIV testing and ARV therapy and ARV PPO. The single indicator with the most divergence from the composite measure was HIV testing at first ANC visit, which was high at all pMTCT clinics (over 95% for half of the study clinics), and thus provided insufficient variability for characterizing high and low-performing pMTCT clinics. Though the proportion of women who received CD4 testing services was low (53.3%), it was included as a performance measurement strategy because at the time of study implementation, CD4 testing was a prerequisite to access care.

### Impact of ranking approach on characteristics associated with performance

Ranking strategy substantially impacted clinic characteristics associated with pMTCT performance (Table 4).

**Table 4:** Clinic characteristics associated with high pMTCT performance by ranking approach

Ranking measure	+Association	*p*	−Association	*p*	No association	*p*
HIV test at first ANC visit & PPO-ART for mother	Catchment size	0.01*	Wait time between PCR blood draw & receipt of results at facility	0.04*	Clinic location (rural, urban, peri-urban)	0.29
	No. MCH nurses	<0.01*			NGO support	0.17
	No. MDs & NPCs	<0.02*			Active tracing LTFU	0.52
	PIMA CD4 analyses at facility	<0.04			HIV+ mothers support group at facility	0.19
	No. of deliveries in prior 6 months	<0.01*			Community linkages	0.43
					Schedule of receiving requisitions & delivering consumables	0.36
					ANC stock outs of:	
					AZT	0.79
					HIV rapid test	0.59
					Pharmacy stock outs of:	
					HIV rapid test	0.79
PPO-ART for mother	Catchment size	<0.01*	Wait time between PCR blood draw and receipt of results at facility	<0.05*	Clinic location (rural, urban, peri-urban)	0.37
	No. of MCH nurses	<0.01*			NGO support	0.16
	No. of MDs & NPCs	0.03*			Active tracing LTFU	0.52
	PIMA CD4 analyses at facility	0.04			Community linkages	0.66
	No. of deliveries in prior 6 months	<0.01*			Schedule of receiving requisitions & delivering consumables	0.57
	HIV+ mothers support group at facility	<0.01			ANC stock outs of:	
					AZT	0.79
					HIV rapid test	0.59
					Pharmacy stock outs of:	
					HIV rapid test	0.38
HIV test at first ANC visit	ANC stock outs of:	0.04	None	NA	Clinic location (rural, urban, peri-urban)	0.42
	AZT				NGO support	0.55
					Active tracing LTFU	0.52
					Community linkages	0.66
					Schedule of receiving requisitions & delivering consumables	0.16
					ANC stock outs of:	
					HIV rapid test	0.59
					Pharmacy stock outs of:	
					HIV rapid test	0.79
					Catchment size	0.83*
					No. of MCH nurses	0.25*
					No. of MDs & NPCs	0.61*
					PIMA CD4 analyses at facility	0.33
					No. of deliveries in prior 6 months	0.80*
					HIV+ mothers support group at facility	0.55
					Wait time between PCR blood draw and receipt of results at facility	0.37*

* All *p*-values are from chi-squared tests unless noted with *(unadjusted logistic regression). PPO: prophylaxis; ART: antiretroviral therapy: ANC: antenatal care; MCH: maternal child health; MD: medical doctors; NPC: non physician clinicians; PIMA CD4: point of care rapid CD4; NGO: non-governmental organization; LTFU: lost to follow up; AZT: Zidovudine.

Using the two variable performance ranking approaches (HIV test at first ANC visit and ARV PPO or cART for mother), five factors were found to be significantly associated with higher pMTCT performance, including larger catchment area, higher number of institutional deliveries, availability of PIMA point-of-care CD4 capacity onsite, higher numbers of MCH nurses and higher number of MDs and NPCs. Longer lag time from polymerase chain reaction (PCR) blood draw to receipt of results at facility was associated with poor pMTCT performance, but shorter lag times were not associated with high-performing pMTCT clinics. Factors not associated with performance included clinic location, availability of NGO support, presence of a women's support group, active community-level patient tracking systems and stock-outs of essential testing supplies and medicines.

Using maternal receipt of ARV PPO or cART as the single indicator for performance ranking, the presence of a support group for people living with HIV was also found to be significantly associated with higher performance; the remaining factors remained unchanged.

Using a HIV test at the first ANC visit as the single indicator for performance ranking dramatically altered the factors associated with pMTCT performance, with only stock-outs of AZT in ANC remaining associated with high pMTCT performance. All other indicators that were positively or negatively associated with pMTCT performance were no longer significant in this analysis.

### Impact of middle-performers on the association of clinic factors and pMTCT performance

Removing the 10 middle-performing clinics and comparing the magnitude of associations with three-level performance did not dramatically alter the results (Table 5).

**Table 5:** Associations of continuous clinic characteristics with performance, using different performance outcomes

	Three-level performance*		Two-level performance (mid-performers omitted)		Two-level performance (low/middle vs. high)		Two-level performance (low vs. middle/high)	
				
Variable	Odds ratio (95% CI)	*p*	Odds ratio (95% CI)	*p*	Odds ratio (95% CI)	*p*	Odds ratio (95% CI)	*p*
Catchment area (per 10,000 inhabitants)	1.92 (1.09–3.38)	0.02	3.57 (1.84–6.91)	0.0002	1.47 (0.92–2.33)	0.10	4.74 (1.46–15.36)	0.01
Wait time (days) between PCR blood draw & receipt of result at clinic	0.95 (0.90–1.00)	0.04	0.10 (0.01–1.41)	0.09	0.96 (0.90–1.02)	0.15	0.91 (0.83–1.00)	0.05
Number of MCH nurses	1.12 (1.03–1.22)	0.009	1.26 (1.08–1.47)	0.003	1.08 (0.90–1.02)	0.06	1.21 (1.05–1.41)	0.01
Number of MDs & NPCs	2.21 (1.12–4.39)	0.02	5.16 (1.22–21.86)	0.03	2.00 (0.99, 4.01)	0.05	5.37 (1.31–21.99)	0.02

* PCR: polymerase chain reaction; MCH: maternal child health; MD: medical doctors; NPCs=non-physician clinicians; *OR calculated using ordinal logistic regression with robust standard errors.

With the exception of lag time between PCR blood draw and receipt of results at the pMTCT clinic, all additional explanatory factors remained significantly associated with performance.

Aggregating the middle-performing clinics with the high or low-performing groups slightly impacted the characteristics significantly associated with pMTCT performance. Most notably, catchment area size was less strongly associated with performance when middle-performing clinics were aggregated with the low-performing group, though the strength and direction of the association was similar to the analysis omitting middle-performing clinics. Decreased waiting time between PCR blood draw and receipt of results at the clinic was more significant when the middle-performing facilities were aggregated with the high-performing group compared with the analysis omitting middle-performing facilities. For both the number of MCH nurses and number of MDs/NPCs, there was no meaningful difference in direction and strength of association when the middle-performing clinics were included in the analysis.

## Discussion

In this article, we explore an approach to classify pMTCT performance based on routinely collected health systems data, coupled with additional data collection on modifiable and non-modifiable health systems factors, to identify best practices for pMTCT. We found that using two measures to classify pMTCT performance was preferable to one, though one measure (effective PPO or cART in pregnancy) provided sufficient performance variability to detect associations with health system factors. In general, combining critical individual components into a single composite measure – as part of a dashboard of measures – may facilitate system-level changes by highlighting the need for better care coordination and accountability across multiple providers or services [42]. However, the use of composite metrics in settings with weak health information systems must ensure that individual measures within the composite are based on the strongest evidence available [43].

Classifying high and low performance provided consistent results across ranking measures, though granularity was improved by including middle performers in either the high or low-performing groups. In this analysis, human resource availability (especially MCH nurses), catchment size and utilization were positively associated with effective pMTCT service delivery. A delay in PCR results was negatively associated with pMTCT service delivery. Notably, measures of community linkages were not associated with pMTCT service delivery across the ranking approaches.

This study was limited by its relatively small sample of 30 facilities from one geographic location in one country, which affects the generalizability of the findings. Nonetheless, the methodology of using high and low performance shows promise, providing a framework that can be evaluated in other settings for HIV and non-HIV related services, and can be used to verify and add to the initial list of modifiable and non-modifiable factors associated with pMTCT performance.

There are considerable on-going efforts to evaluate pMTCT programs at the global, country, and community level. These include novel techniques to estimate HIV-free survival using community surveys as well as more traditional health facility level surveys to assess the relationship between facility characteristics and pMTCT coverage across multiple countries [44, 45]. The methodology explored in this paper is meant to complement this existing work by providing health managers with a practical way in which to benchmark quality improvement efforts.

The principal strength of this study is its reliance on routine health information system data coupled with simple data collection that is feasible during routine supervision. By using health facilities as the unit of analysis (rather than individual patient attributes, knowledge, or behaviours), this study delves into broader determinants of effective pMTCT services that are under the control of health system managers, and can be modified to lead to broad improvements in service effectiveness. Furthermore, the simple analysis approach is geared towards district MCH managers to improve data use for pMTCT service redesign and enhancement, relying on strategies that are effective in their service delivery context.

In this study, increased human resources were significantly associated with pMTCT performance, which is consistent with literature that has highlighted the importance of human resources for better health systems outputs and measures of population health impact [46, 47]. This relationship is logical given severe personnel constraints, and in the context of improving pMTCT service quality, underscores the need for more efficient allocation of limited human resources and significant investments to produce and retain human resources. Thus, any intervention to improve pMTCT delivery must address human resource constraints, either directly, or more likely, indirectly through the introduction of systems analysis and improvement approaches to increase the efficiency of delivery. The lack of associations between pMTCT performance and community and social support may be the result of the sample size, or the overwhelming importance of other factors like personnel availability.

The inability of individual measures (especially HIV testing in pregnancy) to capture a “high-performing” facility was likely due to insufficient variability in this measure across study facilities. Further research into the variability of pMTCT cascade measures in other settings is warranted to guide application of the ranking approach.

The use of routine health information system data to classify health facility pMTCT performance represents an opportunity and a challenge in low resource, high-burden settings such as Mozambique. Using existing data sources reinforces routine information systems and can lead to investment by health workers in assessing and improving service delivery, though incomplete or inconsistent data can result in inconsistent rankings (such as CD4 data, which incidentally will become a less important gateway-to-care measure in decisions about patient care as Option B+ is introduced).

## Conclusions

This study supports using fewer, targeted performance measures for systems analysis. As reporting requirements increase to meet the expectations of funders, less time is available to use data for program improvements by district and frontline health managers. Targeting high and low performance is one practical approach to rapidly use routine pMTCT cascade data to achieve a systems perspective of pMTCT functioning at the health facility level, and to exploit differences across facilities to identify best practices across health facilities to inform system improvement efforts.
